# A novel organotypic cortical slice culture model for traumatic brain injury: molecular changes induced by injury and mesenchymal stromal cell secretome treatment

**DOI:** 10.3389/fncel.2023.1217987

**Published:** 2023-07-18

**Authors:** Francesca Pischiutta, Helena Cavaleiro, Enrico Caruso, Francesca Tribuzio, Noemi Di Marzo, Federico Moro, Firas Kobeissy, Kevin K. Wang, António J. Salgado, Elisa R. Zanier

**Affiliations:** ^1^Department of Acute Brain and Cardiovascular Injury, Istituto di Ricerche Farmacologiche Mario Negri IRCCS, Milan, Italy; ^2^Life and Health Sciences Research Institute (ICVS), School of Medicine, University of Minho, Braga, Portugal; ^3^ICVS/3B’s–PT Government Associate Laboratory, Braga/Guimarães, Portugal; ^4^Neuroscience Intensive Care Unit, Department of Anesthesia and Critical Care, Fondazione IRCCS Cà Granda Ospedale Maggiore Policlinico, Milan, Italy; ^5^Centro Ricerca Tettamanti, Clinica Pediatrica, Università di Milano-Bicocca, Fondazione MBBM, Monza, Italy; ^6^Program for Neurotrauma, Neuroproteomics and Biomarkers Research, Departments of Emergency Medicine, Psychiatry, Neuroscience and Chemistry, University of Florida, Gainesville, FL, United States; ^7^Department of Neurobiology, Center for Neurotrauma, Multiomics and Biomarkers (CNMB), Neuroscience Institute, Morehouse School of Medicine, Atlanta, GA, United States

**Keywords:** traumatic brain injury, 3D *in vitro* model, organotypic cortical brain slices, mesenchymal stromal cells, secretome

## Abstract

Traumatic brain injury (TBI) is a major worldwide neurological disorder with no neuroprotective treatment available. Three-dimensional (3D) *in vitro* models of brain contusion serving as a screening platform for drug testing are lacking. Here we developed a new *in vitro* model of brain contusion on organotypic cortical brain slices and tested its responsiveness to mesenchymal stromal cell (MSC) derived secretome. A focal TBI was induced on organotypic slices by an electromagnetic impactor. Compared to control condition, a temporal increase in cell death was observed after TBI by propidium iodide incorporation and lactate dehydrogenase release assays up to 48 h post-injury. TBI induced gross neuronal loss in the lesion core, with disruption of neuronal arborizations measured by microtubule-associated protein-2 (MAP-2) immunostaining and associated with *MAP-2* gene down-regulation. Neuronal damage was confirmed by increased levels of neurofilament light chain (NfL), microtubule associated protein (Tau) and ubiquitin C-terminal hydrolase L1 (UCH-L1) released into the culture medium 48 h after TBI. We detected glial activation with microglia cells acquiring an amoeboid shape with less ramified morphology in the contusion core. MSC-secretome treatment, delivered 1 h post-injury, reduced cell death in the contusion core, decreased NfL release in the culture media, promoted neuronal reorganization and improved microglia survival/activation. Our 3D *in vitro* model of brain contusion recapitulates key features of TBI pathology. We showed protective effects of MSC-secretome, suggesting the model stands as a tractable medium/high throughput, ethically viable, and pathomimetic biological asset for testing new cell-based therapies.

## Introduction

Traumatic brain injury (TBI) is defined as an alteration in brain function, or other evidence of brain pathology, caused by an external force (Menon et al., 2010). About 50–60 million people sustain a TBI each year, with a high impact on individuals, their families, and the society (GBD 2016 Traumatic Brain Injury and Spinal Cord Injury Collaborators, 2019; Maas et al., 2022). There have been advances in the clinical management of TBI patients, but no neuroprotective or regenerative drugs are available to prevent or mitigate injury progression and chronic neurological deficits.

Animal models have been successful in identifying pathological mechanisms induced by TBI and they are a fundamental step for therapeutic development. However, 3D *in vitro* models recapitulating brain structural complexity are needed as a viable platform for drug screening to address the need to replace, reduce, and refine animal testing (Neuhaus et al., 2022; Wadman, 2023). Organotypic brain slices fit these features well, preserving anatomical architecture, cellular diversity and connectivity, as well as extracellular matrix composition (Nogueira et al., 2022). So far, organotypic hippocampal brain slices have been used to model TBI by stretch (Di Pietro et al., 2013; Dollé et al., 2013; Kang et al., 2015; Omelchenko et al., 2019), blast (Vogel et al., 2016, 2017b) or compressive injuries (Adamchik et al., 2000; Adembri et al., 2008; Coburn et al., 2008; Harris et al., 2013). However, a purely contusive model on cortical areas mimicking a TBI focal lesion is still missing. Thus, we developed a new *in vitro* model of brain contusion by exposing murine cortical organotypic slices to a controlled impact (CCI) delivered by an electromagnetic impactor as largely characterized for *in vivo* TBI studies in rodents.

*In vivo* TBI studies across a range of different models and injury severities show that mesenchymal stromal cells (MSCs) improve functional outcome and anatomical damage (Pischiutta et al., 2021). The pleiotropic protective and reparative events induced are mainly mediated by released bioactive factors contained in the MSC-secretome (Tajiri et al., 2014; Mendes-Pinheiro et al., 2019; Pischiutta et al., 2022). We previously found a protective effect of MSC-secretome on ischemic organotypic brain slices (Pischiutta et al., 2016) and here we tested its activity after a biomechanical impact. We observed that MSC-secretome treatment reduced cell death, restored the network complexity, and promoted microglia response, providing evidence for its application as an effective tool for drug testing.

## Materials and methods

### Study approval

The IRFMN adheres to the principles set out in the following laws, regulations, and policies governing the care and use of laboratory animals: Italian Governing Law (D.lgs 26/2014; Authorisation no. 19/2008-A issued March 6, 2008 by Ministry of Health); Mario Negri Institutional Regulations and Policies providing internal authorization for people conducting animal experiments (Quality Management System Certificate–UNI EN ISO 9001:2008–Reg. N° 8576-A); the NIH Guide for the Care and Use of Laboratory Animals (2011 edition) and EU directives and guidelines (EEC Council Directive 2010/63/UE). They were reviewed and approved by the Mario Negri Institute Animal Care and Use Committee which includes *ad hoc* members for ethical issues and by the Italian Ministry of Health (Decreto no. D/07/2013-B).

C57BL/6J mice were bred (one male and two females per cage) in a specific pathogen free vivarium at a constant temperature (21 ± 1°C) and relative humidity (60 ± 5%), with a 12 h light–dark cycle and *ad libitum* access to food and water. All efforts were made to minimize animal suffering and to reduce the number of animals used.

### Prefrontal cortex slices collection

Organotypic cortical brain slices were obtained under sterile conditions from the prefrontal cortex of C57BL/6J or Cx3Cr^+/GFP^ (expressing green fluorescent protein in microglial cells) mouse pups (P1-P3), as previously described (Dossi et al., 2013; Pischiutta et al., 2016) and as represented in Supplementary Figure 1. Briefly, the brain was removed from the skull and embedded into an agarose solution (3% w/V, Promega). Agarose blocks containing mesencephalic and forebrain areas were fixed onto the specimen stage of a vibratome (Leica, VT 1000S), with super glue. The fixed brain was kept in ice-cold (4°C) artificial cerebral spinal fluid (ASCF, NaCl = 87 mM, NaHCO_3_ = 25 mM, NaH_2_PO_4_ = 1.25 mM, MgCl_2_ = 7 mM, CaCl_2_ = 0.5 mM, KCl = 2.5 mM, D-glucose = 25 mM, sucrose = 75 mM, Penicillin = 50 U/ml, Streptomicin = 50 μg/ml) and oxygenated with 95% of oxygen and 5% CO_2_. Prefrontal cortex slices with 200 μm of thickness were cut and transferred onto petri dishes with cold ACSF (4°C). Intact slices were transferred to membranes of tissue culture inserts (Millicell Culture insert, 0.4 μm pore size, Merck-Millipore). The slices were maintained in culture (37°C, 5% CO_2_), two by two per insert in a 6-well plate. Each well was filled with 1 ml of culture medium [MEM-Glutamax 25%, basal medium eagle 25% (Invitrogen), horse serum 25% (Euroclone), glucose 0.6%, Penicillin 100 U/ml, Streptomicin 100 μg/mL (Euroclone); pH = 7.2] for 2 days, and then cultured in neurobasal medium supplemented with B27 (NB/B27, B27 1:50, L-glutamine 1:100, penicillin 100 U/mL, streptomycin 100 μg/mL) with medium changes every other day.

### *In vitro* CCI

**FIGURE 1:**
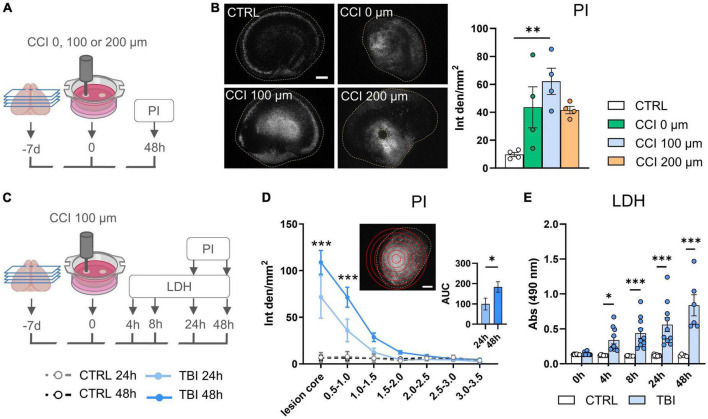
*In vitro* TBI model set-up. **(A)** Experimental design of TBI grading by increasing CCI deformation depth from 0 to 200 μm. **(B)** Representative images and quantification of PI incorporation at 48 h in CTRL and TBI slices. **(C)** Experimental design for spatio/temporal cell death characterization after TBI. **(D)** Quantification of regional-specific PI incorporation on concentric ROIs (top insert) and as AUC (bottom insert). **(E)** Temporal LDH release in culture medium after TBI. Data are mean ± SEM; **(B)** One-way ANOVA followed by Tukey’s multiple comparisons test; **(D,E)** Two-way ANOVA followed by Tukey’s multiple comparison test; *p < 0.05, **p < 0.01, ***p < 0.001. **(D)** *n* = 6–8. **(B–D)** bars = 500 μm. Drawings created with BioRender.com.

After 1 week in culture, cortical slices were subjected to controlled cortical impact (CCI) delivered by an electromagnetic impactor device (Impact One^®^, Leica, USA) to model TBI. A stereotactic apparatus, connected to the impactor was used to set the trauma depth. Each insert with slices was taken from the plate and placed under the impactor, with the proper medium supply. The damage was driven by a 1 mm diameter impactor tip, at 1.5 m/s of speed and 0.1 sec dwell time. At first, we tested three different deformation depths: 0 μm (the piston just touched the slice, without penetrating it), 100 μm or 200 μm, the latter corresponding to the thickness of the whole slice (experimental design in Figure 1A). The 100 μm depth induced a significant increase in PI incorporation with no tissue perforation, thus it was selected for the next experiments (experimental design in Figure 1C). Control slices were subjected to the same protocol, without injury.

### Propidium iodide incorporation

Controlled cortical impact induced cell death was measured by propidium iodide (PI) incorporation assay at tissue level. At 24 h and 48 h after injury, slices were incubated with PI (2 μM diluted in NB/B27) for 30 min, washed with PBS and photographed using an Olympus IX71 inverted microscope. Fluorescence intensity per slice was measured using ImageJ’s Integrated Density function and the value was normalized over the slice area. In order to evaluate cell death in a region-specific manner, the fluorescence intensity was also evaluated in specific regions of interest (ROIs), drawn in a concentric manner, spaced 0.5 mm, starting on the lesion site to the slice periphery.

### Lactate dehydrogenase assay

Cell death was evaluated by a Lactate dehydrogenase (LDH) assay in the culture medium collected at time 0 (before injury), 4, 8, 24, and 48 h after injury and stored at −20°C. LDH release was measured using Cytotox 96 Non-Radioactive Assay (Promega) following manufacturer instructions. Absorbance was read at 490 nm (TECAN, Infinit^®^200 PRO).

### Quantification of injury biomarkers released in the culture medium

To assess neuronal damage, we quantified the release of three different neuronal markers in the culture medium: the neurofilament light chain (NfL), the tubulin associated unit (Tau) and the Ubiquitin C-terminal hydrolase L1 (UCH-L1). Injury to astrocytes was assessed by measuring the release of the glial fibrillary acidic protein (GFAP). Culture medium was collected 48 h post-injury, and stored at −20°C. Biomarker releases in the culture media were measured by single molecule array (simoa) immunoassay (Quanterix, Billerica, MA, USA). Analyses were run using commercial simoa assays on a SR-X Analyzer: NfL advantage assay (catalog #103400) and Neurology 4-Plex B Kit (catalog # 103345). Samples were diluted 1:200 in diluent buffer. A single lot of reagents was used for all samples. All samples were examined in duplicate.

### Immunofluorescence

Forty-eight hours post-CCI, the slices were fixed 1 h in paraformaldehyde (PFA) 4%, dehydrated two overnights in a 30% sucrose solution, rapidly frozen in N-pentane (−45°C) and stored at −80°C. For immunofluorescence staining, the slices were detached from the transwell and incubated overnight in free-floating with the primary antibodies: anti microtubule-associated protein (MAP-2, 1:500 Abcam, ab11267), glial fibrillary acidic protein (GFAP, 1:2000, MERCK, AB5804), and ionized calcium binding adaptor molecule 1 (Iba-1, 1:1000, Wako, 019-19741), followed by 1 h incubation with secondary antibodies anti-mouse alexa Fluor^®^488, or anti-rabbit alexa Fluor ^®^594 (1:500, Invitrogen).

### Confocal microscopy and digital image analysis

**FIGURE 2:**
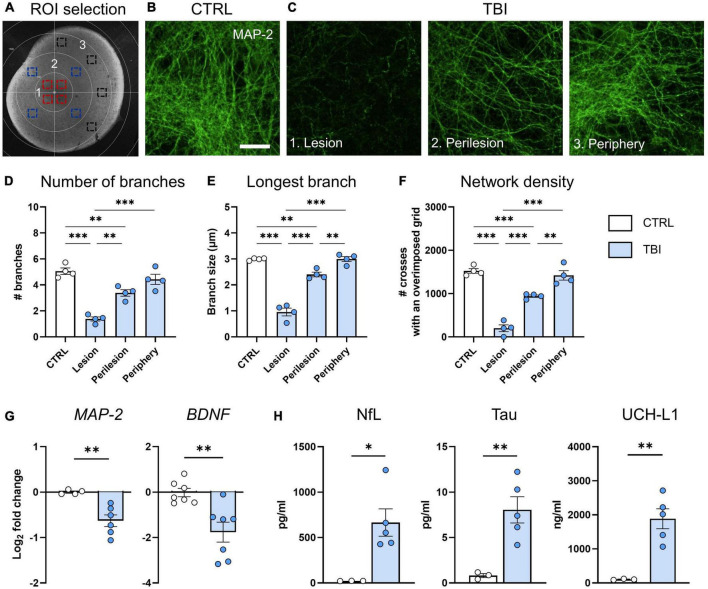
Evaluation of TBI induced neuronal damage 48 h after injury. **(A)** Representative image of ROIs in the lesion (1, red squares), perilesion (2, blue squares) and periphery (3, black squares). Since MAP2 density was uniform in CTRL slices throughout concentric areas, results are shown as the average of the 12 ROIs. **(B)** Representative confocal images of MAP-2 staining in CTRL slices and **(C)** in the lesion, perilesion, and periphery of TBI slices. **(D)** Quantification of the number of neurite branches, **(E)** the size of the longest branch and **(F)** the network density of MAP-2 immunostaining. **(G)** Gene expression analysis of neuronal-associated *MAP-2* and *BDNF* markers in cortical slices. **(H)** Levels of neuron-associated biomarkers released in culture media. Data are mean ± SEM. **(D–F)** One-way ANOVA, followed by Tukey’s multiple comparisons test, **(G,H)** *t*-test. *p < 0.05, ^**^p < 0.01, ^***^p < 0.001. Bar = 25 μm.

Confocal microscopy was done on a Nikon A1 confocal scan unit, managed by NIS elements software. Whole cortical brain slices were imaged at laser excitations of 488 or 594 nm with a sequential scanning mode to avoid bleed-through effects. An overview was obtained with a 10 × 0.5 NA and a concentric grid centered in the lesion site and distanced 500 μm was used to define concentric areas: the lesion, perilesion and periphery (Figure 2A). Within each area, 4 ROIs were acquired with a 40 × 0.75 NA objective. Each image was 1,024 × 1,024, having a pixel size of 0.21 μm and a step size of 0.85 μm for MAP-2 and a pixel size of 0.31 μm and a step size of 1 μm for GFAP and Iba-1. In order to standardize the image acquisition and minimize inter-sample variation, all acquisition parameters were kept constant throughout microscopy sessions.

We quantified the complexity of neuronal arborizations in terms of number, size and density of branches on MAP-2 stained slices, by using an originally developed ImageJ algorithm to segment and skeletonize the signal as previously described (De Paola et al., 2023). Briefly, the background was normalized imposing a minimum gray value of 1,200, and the image was corrected with a median filter with a radius of 5 and binarized over the z stack by the Li method (Li and Tam, 1998). An extended focus image of the binary image was obtained and the skeletonize function was applied. Then the ‘Analyze Skeleton 2D/3D’ ImageJ plugin was applied with no endpoint pruning.

Glial fibrillary acidic protein and IBA1% stained area was measured on 5 focal plans using image J. Microglia morphology was analyzed, as described (Zanier et al., 2015; Moro et al., 2021). A size threshold (40–500 μm^2^) was used to select cells to be analyzed for the following shape descriptors: area, perimeter, Feret’s diameter and circularity. Mean single-cell values for each parameter were used for statistics.

Results within lesion, perilesion and periphery are shown as the mean values of the 4 ROIs. Since no differences were present between concentric areas in CTRL slices, results are shown as the average of the 12 ROIs.

### Gene expression analysis

Forty-eight hours after CCI, slices were collected, and total RNA was extracted by using the Ribospin II extraction kit (GeneAll) following the manufacturer’s protocol. The RNA concentration was determined with a Nanodrop spectrophotometer and 200 ng/μl RNA from each sample was reverse transcribed to cDNA using TaqMan^®^ qPCR RT Master Mix (Applied Biosystems by life technologies) following the manufacturer’s protocol. Real-time RT-PCR was performed, and the relative gene expression to the reference gene *RPL27* was determined by ΔΔCt method. Data are expressed as log_2_ of the fold difference from the CTRL group. The primers used are listed in Table 1. Primers were designed using the Primer-BLAST tool (NCBI, United States),^1^ and Prime3 open-source software.^2^

**TABLE 1:** Primers used for real-time reverse transcription polymerase chain reaction.

	NCBI reference	Forward primer	Reverse primer
RPL27	NM_011289.3	TCATGAAACCCGGGAAAGT	GAGGTGCCATCGTCAATGT
MAP2	NM_0010399341	TCAGCTGACAGAGAAACAGCA	TTGTGTTGGGCTTCCTTCTC
GFAP	NM_001131020	GAAACCGCATCACCATTCC	TCGGATGGAGGTTGGAGA
CD11b	NM_010562.2	GAGCAGCACTGAGATCCTGTTTAA	ATACGACTCCTGCCCTGGAA
BDNF	NM_007540	AGGCACTGGAACTCG	AAGGGCCCGAACATAC

### Secretome treatment

Mesenchymal stromal cells (MSCs) were isolated from human umbilical cord perivascular cells (Fraga et al., 2013) and the secretome was collected as a conditioned medium in passage 8 (P8) as previously described (Mendes-Pinheiro et al., 2019). Briefly, cells were seeded at a density of 4,000 cells/cm^2^ and incubated with α-MEM medium (Gibco, USA), supplemented with 5% human platelet lysate (HPL) 1% penicillin-streptomycin, 0,04% heparin. After 3 days in culture, cells were washed 4 times with PBS without Ca^2+/^Mg^2+^ (Invitrogen) and incubated for 24 h with Neurobasal-A medium (Gibco, USA) supplemented with 1% of penicillin-streptomycin (Fraga et al., 2013). On the next day, the conditioned medium containing the MSC-secretome was collected and centrifuged at 1,200 rpm (Thermo Electron Corporation, EUA) for 5 min to remove any cell debris. Then, secretome was snap frozen in liquid nitrogen and stored at −80°C. Before treating the slices, the collected secretome was supplemented with 2% B27 (Euroclone, IT) and 1% L-glutamine (Euroclone, IT), and applied to the organotypic culture. Treatment was applied 1 h post-CCI and no medium changes were performed during the following 48 h.

### Pathway analysis

For the interactome pathway analysis, we used Elsevier’s Pathway Studio version 10.001.^3^ To establish the various relationships among the different altered validated proteins, ResNet Pathways Studio Propriety database was utilized to infer all the interactions. Interactome network was generated using a “direct interaction” algorithm for cellular processes, biological process mapping as well as the proposed pathway interactions.

### Statistical analysis

Cortical slices were allocated to injury and treatments by a list randomizer.^4^ All evaluations were done blinded to injury/treatment status. Data are presented as mean ± SEM. The choice between parametric or non-parametric tests was based on passing the Shapiro–Wilk normality test, and data distribution was inspected by QQ plot. For each experiment, the figure legend reports the statistical analysis of data. For statistical analyses we used GraphPad Prism (GraphPad Software Inc., USA, version 9.2.0). For the pathway analysis, we used Fisher’s statistical test to determine if there are non-random associations between two categorical variables organized by specific relationship (protein interaction and biological process). The algorithm compares the sub-network distribution to the background distribution using a one-sided Mann–Whitney U-Test, and calculates a *p*-value indicating the statistical significance of the difference between two distributions.

## Results

### *In vitro* TBI model set-up: definition of the optimal injury depth

Traumatic brain injury severity was tailored by impacting cortical slices with CCI deformation depth of 0, 100, or 200 μm (Figure 1A). PI incorporation was highest after CCI at 100 μm deformation depth (*p* < 0.01, Figure 1B). CCI at 200 μm depth resulted in the complete perforation of the brain tissue and loss of the lesion core (Figure 1B) leading to lower overall PI integrated density values. Thus, CCI at 100 μm depth was selected for the subsequent experiments (Figure 1C).

### Spatial and temporal characterization of TBI induced cell death

We quantified regional PI incorporation by drawing concentric ROIs centered in the lesion core (Figure 1D). PI incorporation in TBI slices showed the maximum value in the lesion core (*p* < 0.001) and a gradual decrease toward peripheral ROIs at both 24 and 48 h post-injury (Figure 1D). AUC in TBI slices was higher at 48 compared to 24 h (*p* < 0.05) indicating a spatial and temporal increase in cell death (Figure 1D right insert).

After TBI, LDH release progressively increased up to 48 h with a significant difference already observed at 4 h (*p* < 0.05) compared to controls (Figure 1E) thus confirming the increase of cell death over time. CTRL slices showed a low PI incorporation and LDH release uniform over space and time.

### Evaluation of neuronal and glial damage after TBI

To investigate TBI-induced modifications of neuronal cytoarchitecture we quantified MAP-2 immunofluorescent signal on whole-mounted slices at 48 h post injury, selecting ROIs at lesion, perilesion, and periphery (Figure 2A). Compared to CTRL (Figure 2B), TBI slices showed a strong decrease of MAP-2 staining in the lesion and perilesional areas (Figure 2C). MAP-2 morphometric parameters showed that TBI decreased the complexity of neuronal arborizations, with significant reduction in the number of branches (*p* < 0.001, Figure 2D), in the size of the longest branch (*p* < 0.001, Figure 2E) and in the network density (*p* < 0.001, Figure 2F) at the lesion core, while showing values comparable to controls at the periphery. In accordance, gene expression analysis at 48 h indicated a TBI-induced downregulation of *MAP-2* and the neuronal associated growth factor *BDNF* (*p* < 0.01, Figure 2G).

When analyzing culture media, we found a significant increase of NfL (*p* < 0.05), Tau (*p* < 0.01) and UCH-L1 (*p* < 0.01) levels after TBI, confirming neuronal death (Figure 2H).

**FIGURE 3:**
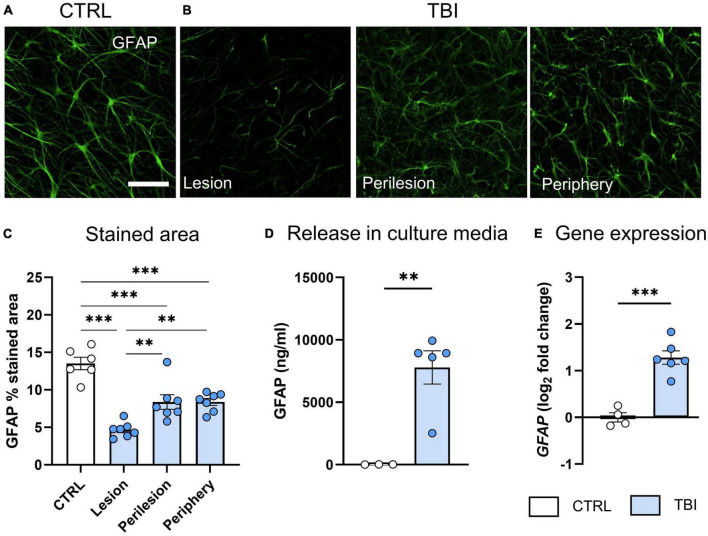
Evaluation of TBI-induced astrocytic damage/activation 48 h after injury. **(A)** Representative confocal images of GFAP staining in CTRL slices and **(B)** in the lesion, perilesion and periphery of TBI slices. Since GFAP staining was uniform in CTRL slices throughout concentric areas, results are shown as the average of the 12 ROIs. **(C)** Quantification of the GFAP% stained area. **(D)** Levels of GFAP biomarker released in culture media. **(E)** Gene expression analysis of *GFAP* marker in cortical slices. Data are mean ± SEM. **(C)** One-way ANOVA, followed by Tukey’s multiple comparisons test, **(D,E)** *t*-test. ^**^p < 0.01, ^***^p < 0.001. Bar = 50 μm.

Histological analysis of GFAP^+^ astrocytic marker, showed a uniform labeling in CTRL slices, cells displayed the typical morphology of cortical astrocytes with defined cell somata and long processes protruding outward (Figure 3A). At 48 h post injury, GFAP stained area significantly decreased in all ROIs (*p* < 0.001), with the highest effect in the lesion core (Figures 3B, C). TBI also induced a significant release of GFAP in culture media 48 h post-injury (*p* < 0.01, Figure 3D), concurrent with an upregulation of *GFAP* gene expression (*p* < 0.001, Figure 3E).

**FIGURE 4:**
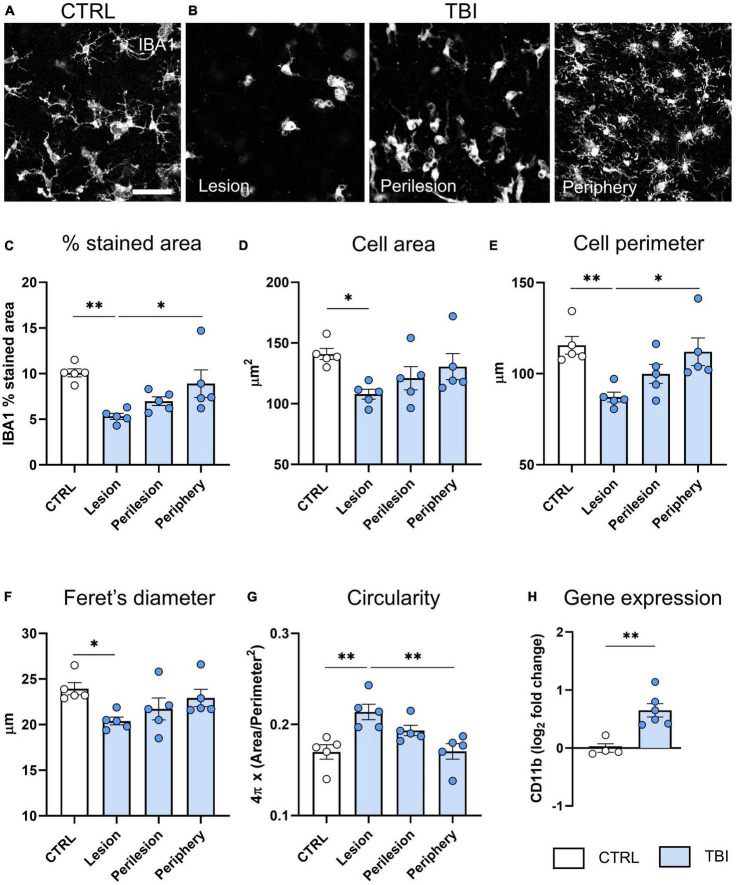
Evaluation of TBI induced microglia changes 48 h after injury. **(A)** Representative confocal images of IBA-1 staining in CTRL slices and **(B)** in the lesion, perilesion, and periphery of TBI slices. Since Iba1 staining was uniform in CTRL slices throughout concentric areas, results are shown as the average of the 12 ROIs. **(C)** Quantification of Iba-1% stained area, and morphometric cell parameters: **(D)** mean cell area, **(E)** perimeter, **(F)** Feret’s diameter and **(G)** circularity. **(H)** Gene expression analysis of microglial *CD11b* marker in cortical slices. Data are mean ± SEM. **(C–G)** One-way ANOVA, followed by Tukey’s multiple comparisons test, **(H)** *t*-test. *p < 0.05 ^**^p < 0.01. Bar = 50 μm.

We found a reduction of IBA1^+^ stained area in the lesion area of TBI slices compared to CTRL (*p* < 0.01, Figures 4A–C) that progressively recovered toward the periphery. Analysis of shape parameters (Figures 4D–G) showed a decreased cell area (*p* < 0.05, Figure 4D), perimeter (*p* < 0.01, Figure 4E) and Feret’s diameter (*p* < 0.05, Figure 4F) in the lesion core of TBI slices, indicating the polarization of microglial cells toward an amoeboid shape with a less ramified morphology. Accordingly, an increase in circularity value was observed in the lesion core (*p* < 0.01, Figure 4G). In the periphery, morphometric parameters were comparable to controls. Gene expression analysis revealed that TBI-induced an upregulation of *CD11b* microglial pan marker (*p* < 0.01, Figure 4H).

### Systems biology analysis

Using the differentially expressed mRNA and proteins released in the culture media, system biology analysis was conducted to identify cellular processes represented in our *in vitro* TBI model. Altered proteins (NfL, Tau, UCH-L1, MAP2, GFAP) were related with other two TBI well established biomarkers (S100-B and neuron specific enolase, NSE) (Supplementary Figure 2). Pathway analysis revealed an association with neuronal death, nerve injury and degeneration, as well as glial reaction and astrocytosis (Supplementary Figure 2).

### MSC-secretome rescues TBI induced damage

**FIGURE 5:**
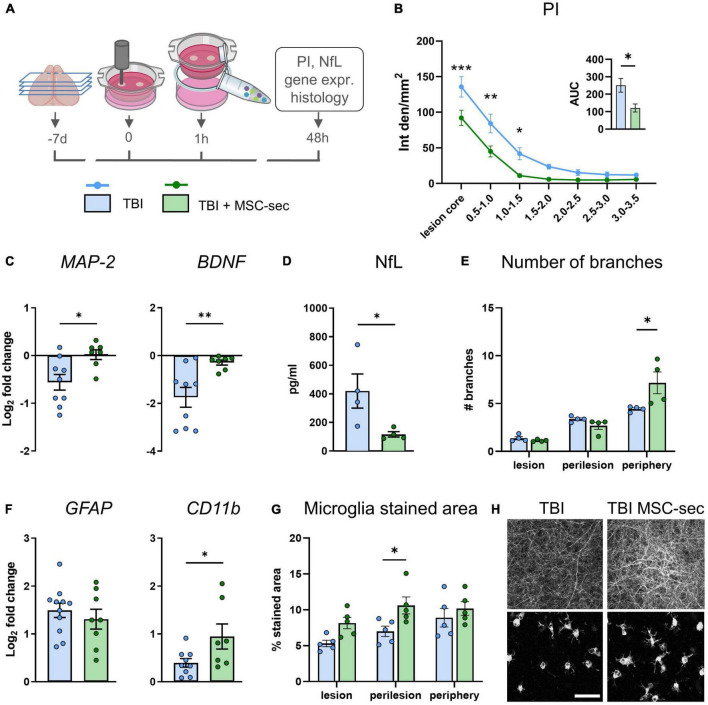
MSC-sec protects brain tissue after TBI. **(A)** Experimental design. **(B)** Quantification of PI incorporation at 48 h after injury in concentric ROIs and as AUC (insert). **(C)** *MAP-2* and *BDNF* gene expression analysis in cortical slices. **(D)** Levels of NfL biomarker released in culture media. **(E)** Histological quantification of the number of neurite branches. **(F)** *GFAP* and *CD11b* gene expression analysis. **(G)** Histological quantification of microglia% stained area. **(H)** Representative confocal images of MAP-2 staining and microglial cells in TBI and MSC-sec treated slices. Data are mean ± SEM. **(B)** Two-way ANOVA, followed by Tukey post test, *n* = 8–10. **(C,D,F)** *t*-test, **(E,G)** Two-way ANOVA, followed by Sidak’s post test, *p < 0.05, ^**^p < 0.01, ^***^p < 0.001. Bar = 50 μm. Drawings created with BioRender.com.

We assessed the effects of MSC-derived secretome administration, delivered to TBI slices 1 h after injury in the culture media (experimental design in Figure 5A). Cell death analysis at 48 h by PI incorporation assay revealed a significant protection induced by MSC-secretome in the lesion core and the adjacent regions (*p* < 0.001, Figure 5B), resulting in a global reduction of cell death (AUC TBI vs TBI + MSC-sec: *p* < 0.01, Figure 5B insert). Analysis of MSC-induced effects on neurons showed a rescue effect on TBI-induced downregulation of *MAP-2* (*p* < 0.05) and *BDNF* (*p* < 0.01, Figure 5C), a decrease of NfL release in the culture media (Figure 5D), and an increase of neurite branches in the periphery of the slice (Figures 5E, H), while no changes were detected for the longest branch size and network density (Supplementary Figure 3). Analysis on glial cells revealed no differences on *GFAP* and an up-regulation of microglial *CD11b* expression (*p* < 0.05) induced by MSC-secretome compared to TBI untreated slices (Figure 5F). Histological analysis of microglia activation revealed an increased stained area in MSC-secretome treated vs untreated TBI slices (Figures 5G, H, Two-way ANOVA p of treatment <0.01), but no changes in shape descriptors (Supplementary Figure 4).

## Discussion

In the present work, we developed a new 3D *in vitro* TBI model on organotypic cortical brain slices subjected to a biomechanical impact (CCI). The model recapitulates key features of *in vivo* CCI in mice, including a focal injury characterized by neuronal, microglial and astrocytic cell death, and the release of neuronal and astrocytic proteins in the cultured media. When treated with MSC-secretome, TBI slices showed reduced cell death and neuronal damage, demonstrating the potential of this model as a valuable tool to test for therapeutic candidates.

Failures in translating preclinical results in the clinical setting call for new experimental models to test therapeutic candidates (Kumaria, 2017). Our work aimed to establishing a reliable *in vitro* TBI model on 3D brain tissue, allowing a close representation of the post-traumatic cellular and molecular events. To date, TBI modeled in organotypic slices used hippocampal tissues (Kang et al., 2015; Effgen and Morrison, 2017; Vogel et al., 2017a), an area that, *in vivo*, is mainly damaged by secondary injury mechanisms (Osier and Dixon, 2016; Siebold et al., 2018). To better reflect the *in vivo* condition, we decided to model brain contusion on cerebral slices obtained from cortices. As impactor, we used the electromagnetic-driven piston, commonly used in *in vivo* TBI studies on rodents. The system has the advantage of a fine control of the impact parameters (velocity, dwell time and deformation depth) and a localized injury, while the existing stretch, blast or compression models provide a more diffuse damage. When tailoring injury severity, 100 μm injury depth induced a consistent focal lesion, with PI accumulating at the lesion core and progressively decreasing toward the periphery. Cell death increased overtime, indicating that brain pathology is progressing, thus mirroring the injury evolution reported in TBI animals (Siebold et al., 2018). By 0 μm deformation depth, with the piston only touching the slice, tissue damage was also observed, likely due to the perturbation air wave during the strike, with high variability and no difference from controls. 200 μm deformation depth destroyed the strike area with clear tissue perforation thus preventing the evaluation of tissue damage.

One of the hallmarks of TBI is neuronal injury, with the disruption of network connections and function. Compared to 2D neuronal cell cultures, the proposed model has a 3D structure preserving the cytoarchitecture of the *in vivo* cerebral microenvironment in terms of cell-cell and cell-extracellular matrix interactions, neuronal network and synaptic organization (Humpel, 2015; Croft et al., 2019; AlaylioĞlu et al., 2020), thus the assessment of neuronal network alterations has translational value. Histological analysis of MAP-2 stained neurites showed an altered network organization after TBI, with a higher effect in the lesion core associated with a reduced number and size of branches and network density. Of note, these parameters follow a gradient distribution with values in the periphery comparable to uninjured slices. Accordingly, gene expression analysis showed *MAP-2* downregulation in conjunction with a decrease of trophic support (*BDNF*). Biomarker evaluation in cell culture media confirmed high neuronal damage, with massive release of proteins from axonal (NfL and tau) and cell body (UCH-L1) structures (Wang et al., 2018). Those are the biomarkers found to be elevated in TBI patients in blood with injury severity dependency (Whitehouse et al., 2022) and showing prognostic value (Shahim et al., 2016; Graham et al., 2021; Helmrich et al., 2022; Korley et al., 2022; Moro et al., 2022) thus supporting the utility of the developed *in vitro* model.

We also explored glial susceptibility/activation. Astrocytes and microglial cells are essential contributions to several homeostatic functions that could directly influence neuronal survival and tissue integrity after TBI (Karve et al., 2016; Michinaga and Koyama, 2021). Histological analysis revealed a TBI-induced astrocytic damage with greater effect in the lesion core. Concordantly, GFAP released in the culture medium indicated astrocyte death. Gene expression analysis showed discordant results, pointing to a *GFAP* mRNA up-regulation after TBI. During highly dynamic adaptation processes, as in response to injury, post-transcriptional events may lead to deviation from an ideal mRNA-protein correlation and a delay timeframe is likely to occur (Liu et al., 2016).

Similar to what was observed in astrocytes, we observed a decrease in microglia stained area within the lesion and a concomitant up-regulation of *CD11b* gene expression. A more detailed analysis of microglia morphometric parameters demonstrated a clear transition from a ramified to an amoeboid cell morphology in the lesion core, as demonstrated by the reduction of cell area, perimeter, Feret’s diameter, and increased circularity. These results recapitulate findings in TBI mice (Zanier et al., 2015; Kumar et al., 2016; Caplan et al., 2020) emphasizing the value of the model. In the peripheral areas, morphometric parameters were not different from control slices.

To establish a link between the *in vitro* and *in vivo* complexity, we used a system biology approach to integrate changes at the protein and gene levels into biological processes. Altered validated proteins (NfL, Tau, UCH-L1, MAP2, GFAP) were studied in relationship with other two established TBI-biomarkers (S100-B and NSE) showing their implication in astrogliosis and astrocytosis, neuronal injury and degeneration, associated with learning and memory deficits (Supplementary Figure 2). This analysis further confirms that our *in vitro* TBI model integrates well into complex biological processes otherwise accessible in *in vivo* TBI models only.

We next tested our model response to the therapeutic efficacy of MSC-based approach. MSCs have shown large beneficial effects in preclinical TBI models (Pischiutta et al., 2021) and now their secreted bioactive factors (i.e., secretome) stand as the main drivers of protection, paving the way for a potential cell-free approach. MSC-secretome treatment of TBI-injured slices induced a reduction of PI incorporation in the lesion core and surrounding regions, pointing to a protection against cell death by MSC-secretome. MSC-secretome induced neuronal protection was confirmed at both gene and protein levels. In particular, MSC-secretome reduced the release of the axonal injury maker NfL in the culture media, providing evidence of the NFL validity as pharmacodynamics biomarker in monitoring treatment response in the TBI model. In addition, histological analysis revealed an increased number of branches in treated slices, in accordance with previous findings from our group, showing MSC-secretome promotion of axonal outgrowth in primary cultures of rat embryonic hippocampal and cortical neurons, with a major role of BDNF in the observed effects (Martins et al., 2017). MSCs have been shown to induce a microglia protective phenotype (Zanier et al., 2011, 2014; Pischiutta et al., 2016, 2022; Kota et al., 2017; Xu et al., 2020) and reduce pyroptosis, a type of programmed cell death associated with inflammatory response (Feng et al., 2022). We found that MSC-secretome promoted microglia activation calling for in-depth analysis of microglia functional state.

Limitations of this model include the lack of a functional vasculature and of systemic infiltrating immune cells playing an important role in the evolution/resolution of brain damage after TBI (Plesnila, 2016). At the same time our model has the major advantage of allowing the specific study of microglial role after TBI, without the presence of infiltrated monocyte/macrophage cells whose distinction from round-shape microglia after *in vivo* TBI is challenging (Jurga et al., 2020). The isolation of brain pathological mechanisms without confounding aspects can help verifying tissue-specific punctual hypothesis.

In addition, our model cannot reproduce the influence of cerebrovascular autoregulation, intracranial pressure and neurovascular coupling (Li et al., 2016), which are critical in TBI pathophysiology, and is not suitable to assess chronic neurodegenerative processes (such as those due to proteinopathies and amyloid accumulations), for which *in vivo* rodents TBI models remains the best options.

Further implementation of the model could include the assessment of the functional neural connections, by a fine characterization of the synaptic integrity and by electrophysiological measurements. In addition, we obtained slices from postnatal donors, because of high survival in culture compared to adult donors (Humpel, 2015). Our method, as described by Dossi et al. (2013) shows a progressive maturation of cortical brain slices reaching a synchronized electrical activity by 5–7 days in culture, comparable to adult mouse brain. The recent set-up of long-term organotypic culture from adult rodents (Humpel, 2019; Mayerl and Ffrench-Constant, 2021) will allow future studies to address the impact of the donor age in injury evolution and response to treatment.

## Conclusion

In the present study, we developed a new TBI model on organotypic cortical slices, a 3D tissue in which the proportion of cerebral populations and their anatomical architecture are representatives of the *in vivo* structure. The injury recapitulates key features of TBI pathology, including neuronal damage, network changes, and glial activation with the possibility to assess injury and therapeutic responses by outcome measurements, with direct translational value, like fluid biomarkers. Our findings provide the first evidence that this new 3D *in vitro* TBI model stands as a tractable medium/high throughput, ethically viable, and pathomimetic biological asset for testing new cell-based therapies.

## Data availability statement

The dataset presented in this study can be found at: https://zenodo.org/badge/DOI/10.5281/zenodo.8046693.svg.

## Ethics statement

The animal study was reviewed and approved by the Mario Negri Institute Animal Care.

## Author contributions

FP and HC performed experiment on the *in vitro* model and analysis and prepared the manuscript. EC, FT, and ND performed histological analysis. FK conducted biomarker quantification and pathway analysis. FM, KKW, and AJS provided a critical review of the manuscript. ERZ designed and supervised the study and reviewed the manuscript. All authors read and approved the final manuscript.

## Footnotes

1
https://www.ncbi.nlm.nih.gov/tools/primer-blast/index.cgi

2
https://primer3.ut.ee/

3
https://www.elsevier.com/solutions/pathway-studio-biological-research

4
https://www.random.org/lists/

## References

[B1] Adamchik Y. Frantseva M. V. Weisspapir M. Carlen P. L. Perez Velazquez J. L. (2000). Methods to induce primary and secondary traumatic damage in organotypic hippocampal slice cultures. *Brain Res. Brain Res. Protoc.* 5 153–158. 10.1016/s1385-299x(00)00007-6 10775835

[B2] Adembri C. Massagrande A. Tani A. Miranda M. Margheri M. De Gaudio R. (2008). Carbamylated erythropoietin is neuroprotective in an experimental model of traumatic brain injury. *Crit. Care Med.* 36 975–978. 10.1097/CCM.0B013E3181644343 18176311

[B3] AlaylioĞlu M. Dursun E. Yilmazer S. Ak D. G. (2020). A bridge between in vitro and in vivo studies in neuroscience: Organotypic brain slice cultures. *Noro Psikiyatr. Ars.* 57 333–337. 10.29399/npa.26139 33354128 PMC7735142

[B4] Caplan H. W. Cardenas F. Gudenkauf F. Zelnick P. Xue H. Cox C. S. (2020). Spatiotemporal distribution of microglia after traumatic brain injury in male mice. *ASN Neuro* 12:1759091420911770. 10.1177/1759091420911770 32146827 PMC7066592

[B5] Coburn M. Maze M. Franks N. P. (2008). The neuroprotective effects of xenon and helium in an in vitro model of traumatic brain injury. *Crit. Care Med.* 36 588–595. 10.1097/01.CCM.0B013E3181611F8A6 18216607

[B6] Croft C. L. Futch H. S. Moore B. D. Golde T. E. (2019). Organotypic brain slice cultures to model neurodegenerative proteinopathies. *Mol. Neurodegener.* 14:45. 10.1186/s13024-019-0346-0 31791377 PMC6889333

[B7] De Paola M. Pischiutta F. Comolli D. Mariani A. Kelk J. Lisi I. (2023). Neural cortical organoids from self-assembling human iPSC as a model to investigate neurotoxicity in brain ischemia. *J. Cereb. Blood Flow Metab.* 43 680–693. 10.1177/0271678X231152023 36655331 PMC10108182

[B8] Di Pietro V. Amorini A. M. Tavazzi B. Hovda D. A. Signoretti S. Giza C. C. (2013). Potentially neuroprotective gene modulation in an in vitro model of mild traumatic brain injury. *Mol. Cell. Biochem.* 375 185–198. 10.1007/s11010-012-1541-2 23242602

[B9] Dollé J.-P. Morrison B. Schloss R. S. Yarmush M. L. (2013). An organotypic uniaxial strain model using microfluidics. *Lab Chip* 13 432–442. 10.1039/c2lc41063j 23233120 PMC3546521

[B10] Dossi E. Heine C. Servettini I. Gullo F. Sygnecka K. Franke H. (2013). Functional regeneration of the ex-vivo reconstructed mesocorticolimbic dopaminergic system. *Cereb. Cortex* 23 2905–2922. 10.1093/cercor/bhs275 22989581

[B11] Effgen G. B. Morrison B. (2017). Memantine reduced cell death, astrogliosis, and functional deficits in an in vitro model of repetitive mild traumatic brain injury. *J. Neurotrauma* 34 934–942. 10.1089/neu.2016.4528 27450515

[B12] Feng Z. Hua S. Li W. Han J. Li F. Chen H. (2022). Mesenchymal stem cells protect against TBI-induced pyroptosis in vivo and in vitro through TSG-6. *Cell Commun. Signal.* 20:125. 10.1186/s12964-022-00931-2 35982465 PMC9387023

[B13] Fraga J. S. Silva N. A. Lourenço A. S. Gonçalves V. Neves N. M. Reis R. L. (2013). Unveiling the effects of the secretome of mesenchymal progenitors from the umbilical cord in different neuronal cell populations. *Biochimie* 95 2297–2303. 10.1016/j.biochi.2013.06.028 23851197

[B14] GBD 2016 Traumatic Brain Injury and Spinal Cord Injury Collaborators (2019). Global, regional, and national burden of traumatic brain injury and spinal cord injury, 1990-2016: A systematic analysis for the Global Burden of Disease Study 2016. *Lancet Neurol.* 18 56–87. 10.1016/S1474-4422(18)30415-0 30497965 PMC6291456

[B15] Graham N. S. N. Zimmerman K. A. Moro F. Heslegrave A. Maillard S. A. Bernini A. (2021). Axonal marker neurofilament light predicts long-term outcomes and progressive neurodegeneration after traumatic brain injury. *Sci. Transl. Med.* 13:eabg9922. 10.1126/scitranslmed.abg9922 34586833

[B16] Harris K. Armstrong S. P. Campos-Pires R. Kiru L. Franks N. P. Dickinson R. (2013). Neuroprotection against traumatic brain injury by xenon, but not argon, is mediated by inhibition at the N-methyl-D-aspartate receptor glycine site. *Anesthesiology* 119 1137–1148. 10.1097/ALN.0b013e3182a2a265 23867231

[B17] Helmrich I. R. A. R. Czeiter E. Amrein K. Büki A. Lingsma H. F. Menon D. K. (2022). Incremental prognostic value of acute serum biomarkers for functional outcome after traumatic brain injury (CENTER-TBI): An observational cohort study. *Lancet Neurol.* 21 792–802. 10.1016/S1474-4422(22)00218-6 35963262

[B18] Humpel C. (2015). Organotypic brain slice cultures: A review. *Neuroscience* 305 86–98. 10.1016/j.neuroscience.2015.07.086 26254240 PMC4699268

[B19] Humpel C. (2019). Organotypic brain slices of ADULT transgenic mice: A tool to study Alzheimer’s disease. *Curr. Alzheimer Res.* 16 172–181. 10.2174/1567205016666181212153138 30543174

[B20] Jurga A. M. Paleczna M. Kuter K. Z. (2020). Overview of general and discriminating markers of differential microglia phenotypes. *Front. Cell. Neurosci.* 14:198. 10.3389/fncel.2020.00198 32848611 PMC7424058

[B21] Kang W. H. Cao W. Graudejus O. Patel T. P. Wagner S. Meaney D. F. (2015). Alterations in hippocampal network activity after in vitro traumatic brain injury. *J. Neurotrauma* 32 1011–1019. 10.1089/neu.2014.3667 25517970 PMC5685195

[B22] Karve I. P. Taylor J. M. Crack P. J. (2016). The contribution of astrocytes and microglia to traumatic brain injury. *Br. J. Pharmacol.* 173 692–702. 10.1111/bph.13125 25752446 PMC4742296

[B23] Korley F. K. Jain S. Sun X. Puccio A. M. Yue J. K. Gardner R. C. (2022). Prognostic value of day-of-injury plasma GFAP and UCH-L1 concentrations for predicting functional recovery after traumatic brain injury in patients from the US TRACK-TBI cohort: An observational cohort study. *Lancet Neurol.* 21 803–813. 10.1016/S1474-4422(22)00256-3 35963263 PMC9462598

[B24] Kota D. J. Prabhakara K. S. Toledano-Furman N. Bhattarai D. Chen Q. DiCarlo B. (2017). Prostaglandin E2 indicates therapeutic efficacy of mesenchymal stem cells in experimental traumatic brain injury. *Stem Cells* 35 1416–1430. 10.1002/stem.2603 28233425

[B25] Kumar A. Barrett J. P. Alvarez-Croda D.-M. Stoica B. A. Faden A. I. Loane D. J. (2016). NOX2 drives M1-like microglial/macrophage activation and neurodegeneration following experimental traumatic brain injury. *Brain Behav. Immun.* 58 291–309. 10.1016/j.bbi.2016.07.158 27477920 PMC5067217

[B26] Kumaria A. (2017). In vitro models as a platform to investigate traumatic brain injury. *Altern. Lab. Anim.* 45 201–211. 10.1177/026119291704500405 28994300

[B27] Li C. H. Tam P. K. S. (1998). An iterative algorithm for minimum cross entropy thresholding. *Pattern Recognit. Lett.* 19 771–776. 10.1016/S0167-8655(98)00057-9

[B28] Li Q. Han X. Wang J. (2016). Organotypic hippocampal slices as models for stroke and traumatic brain injury. *Mol. Neurobiol.* 53 4226–4237. 10.1007/s12035-015-9362-4 26223803 PMC4733438

[B29] Liu Y. Beyer A. Aebersold R. (2016). On the dependency of cellular protein levels on mRNA abundance. *Cell* 165 535–550. 10.1016/j.cell.2016.03.014 27104977

[B30] Maas A. I. R. Menon D. K. Manley G. T. Abrams M. Åkerlund C. Andelic N. (2022). Traumatic brain injury: Progress and challenges in prevention, clinical care, and research. *Lancet Neurol.* 21 1004–1060. 10.1016/S1474-4422(22)00309-X 36183712 PMC10427240

[B31] Martins L. F. Costa R. O. Pedro J. R. Aguiar P. Serra S. C. Teixeira F. G. (2017). Mesenchymal stem cells secretome-induced axonal outgrowth is mediated by BDNF. *Sci. Rep.* 7:4153. 10.1038/s41598-017-03592-1 28646200 PMC5482809

[B32] Mayerl S. Ffrench-Constant C. (2021). Establishing an adult mouse brain hippocampal organotypic slice culture system that allows for tracing and pharmacological manipulation of ex vivo neurogenesis. *Bio Protoc.* 11:e3869. 10.21769/BioProtoc.3869 33732759 PMC7952928

[B33] Mendes-Pinheiro B. Anjo S. I. Manadas B. Da Silva J. D. Marote A. Behie L. A. (2019). Bone marrow mesenchymal stem cells’ secretome exerts neuroprotective effects in a Parkinson’s disease rat model. *Front. Bioeng. Biotechnol.* 7:294. 10.3389/fbioe.2019.00294 31737616 PMC6838134

[B34] Menon D. K. Schwab K. Wright D. W. Maas A. I. Demographics and Clinical Assessment Working Group of the International and Interagency Initiative toward Common Data Elements for Research on Traumatic Brain Injury and Psychological Health (2010). Position statement: Definition of traumatic brain injury. *Arch. Phys. Med. Rehabil.* 91 1637–1640. 10.1016/j.apmr.2010.05.017 21044706

[B35] Michinaga S. Koyama Y. (2021). Pathophysiological responses and roles of astrocytes in traumatic brain injury. *Int. J. Mol. Sci.* 22:6418. 10.3390/ijms22126418 34203960 PMC8232783

[B36] Moro F. Fossi F. Magliocca A. Pascente R. Sammali E. Baldini F. (2021). Efficacy of acute administration of inhaled argon on traumatic brain injury in mice. *Br. J. Anaesth.* 126 256–264. 32977957 10.1016/j.bja.2020.08.027

[B37] Moro F. Lisi I. Tolomeo D. Vegliante G. Pascente R. Mazzone E. (2022). Acute blood levels of neurofilament light indicate one-year white matter pathology and functional impairment in repetitive mild traumatic brain injured mice. *J. Neurotrauma* 40 1144–1163. 10.1089/neu.2022.0252 36576018

[B38] Neuhaus W. Reininger-Gutmann B. Rinner B. Plasenzotti R. Wilflingseder D. De Kock J. (2022). The current status and work of three rs centres and platforms in Europe. *Altern. Lab. Anim.* 50 381–413. 10.1177/02611929221140909 36458800

[B39] Nogueira G. O. Garcez P. P. Bardy C. Cunningham M. O. Sebollela A. (2022). Modeling the human brain with ex vivo slices and in vitro organoids for translational neuroscience. *Front. Neurosci.* 16:838594. 10.3389/fnins.2022.838594 35281505 PMC8908416

[B40] Omelchenko A. Shrirao A. B. Bhattiprolu A. K. Zahn J. D. Schloss R. S. Dickson S. (2019). Dynamin and reverse-mode sodium calcium exchanger blockade confers neuroprotection from diffuse axonal injury. *Cell Death Dis.* 10:727. 10.1038/s41419-019-1908-3 31562294 PMC6765020

[B41] Osier N. D. Dixon C. E. (2016). The controlled cortical impact model: Applications, considerations for researchers, and future directions. *Front. Neurol.* 7:134. 10.3389/fneur.2016.00134 27582726 PMC4987613

[B42] Pischiutta F. Brunelli L. Romele P. Silini A. Sammali E. Paracchini L. (2016). Protection of brain injury by amniotic mesenchymal stromal cell-secreted metabolites. *Crit. Care Med.* 44 e1118–e1131. 10.1097/CCM.0000000000001864 27441900

[B43] Pischiutta F. Caruso E. Cavaleiro H. Salgado A. J. Loane D. J. Zanier E. R. (2022). Mesenchymal stromal cell secretome for traumatic brain injury: Focus on immunomodulatory action. *Exp. Neurol.* 357:114199. 10.1016/j.expneurol.2022.114199 35952763

[B44] Pischiutta F. Caruso E. Lugo A. Cavaleiro H. Stocchetti N. Citerio G. (2021). Systematic review and meta-analysis of preclinical studies testing mesenchymal stromal cells for traumatic brain injury. *NPJ Regen. Med.* 6:71. 10.1038/s41536-021-00182-8 34716332 PMC8556393

[B45] Plesnila N. (2016). The immune system in traumatic brain injury. *Curr. Opin. Pharmacol.* 26 110–117. 10.1016/j.coph.2015.10.008 26613129

[B46] Shahim P. Gren M. Liman V. Andreasson U. Norgren N. Tegner Y. (2016). Serum neurofilament light protein predicts clinical outcome in traumatic brain injury. *Sci. Rep.* 6:36791. 10.1038/srep36791 27819296 PMC5098187

[B47] Siebold L. Obenaus A. Goyal R. (2018). Criteria to define mild, moderate, and severe traumatic brain injury in the mouse controlled cortical impact model. *Exp. Neurol.* 310 48–57. 10.1016/j.expneurol.2018.07.004 30017882

[B48] Tajiri N. Acosta S. A. Shahaduzzaman M. Ishikawa H. Shinozuka K. Pabon M. (2014). Intravenous transplants of human adipose-derived stem cell protect the brain from traumatic brain injury-induced neurodegeneration and motor and cognitive impairments: Cell graft biodistribution and soluble factors in young and aged rats. *J. Neurosci.* 34 313–326. 10.1523/JNEUROSCI.2425-13.2014 24381292 PMC3866490

[B49] Vogel E. W. Effgen G. B. Patel T. P. Meaney D. F. Bass C. R. D. Morrison B. (2016). Isolated primary blast inhibits long-term potentiation in organotypic hippocampal slice cultures. *J. Neurotrauma* 33 652–661. 10.1089/neu.2015.4045 26414012 PMC5583564

[B50] Vogel E. W. Rwema S. H. Meaney D. F. Bass C. R. D. Morrison B. (2017b). Primary blast injury depressed hippocampal long-term potentiation through disruption of synaptic proteins. *J. Neurotrauma* 34 1063–1073. 10.1089/neu.2016.4578 27573357

[B51] Vogel E. W. Morales F. N. Meaney D. F. Bass C. R. Morrison B. (2017a). Phosphodiesterase-4 inhibition restored hippocampal long term potentiation after primary blast. *Exp. Neurol.* 293 91–100. 10.1016/j.expneurol.2017.03.025 28366471 PMC6016024

[B52] Wadman M. (2023). *FDA no longer needs to require animal tests before human drug trials.* Available online at: https://www.science.org/content/article/fda-no-longer-needs-require-animal-tests-human-drug-trials (accessed January 25, 2023).

[B53] Wang K. K. Yang Z. Zhu T. Shi Y. Rubenstein R. Tyndall J. A. (2018). An update on diagnostic and prognostic biomarkers for traumatic brain injury. *Expert Rev. Mol. Diagn.* 18 165–180. 10.1080/14737159.2018.1428089 29338452 PMC6359936

[B54] Whitehouse D. P. Monteiro M. Czeiter E. Vyvere T. V. Valerio F. Ye Z. (2022). Relationship of admission blood proteomic biomarkers levels to lesion type and lesion burden in traumatic brain injury: A CENTER-TBI study. *EBioMedicine* 75:103777. 10.1016/j.ebiom.2021.103777 34959133 PMC8718895

[B55] Xu C. Diao Y.-F. Wang J. Liang J. Xu H.-H. Zhao M.-L. (2020). Intravenously infusing the secretome of adipose-derived mesenchymal stem cells ameliorates neuroinflammation and neurological functioning after traumatic brain injury. *Stem Cells Dev.* 29 222–234. 10.1089/scd.2019.0173 31830866

[B56] Zanier E. R. Fumagalli S. Perego C. Pischiutta F. De Simoni M.-G. (2015). Shape descriptors of the “never resting” microglia in three different acute brain injury models in mice. *Intensive Care Med. Exp.* 3:39. 10.1186/s40635-015-0039-0 26215806 PMC4513020

[B57] Zanier E. R. Montinaro M. Vigano M. Villa P. Fumagalli S. Pischiutta F. (2011). Human umbilical cord blood mesenchymal stem cells protect mice brain after trauma. *Crit. Care Med.* 39 2501–2510. 10.1097/CCM.0b013e31822629ba 21725237

[B58] Zanier E. R. Pischiutta F. Riganti L. Marchesi F. Turola E. Fumagalli S. (2014). Bone marrow mesenchymal stromal cells drive protective M2 microglia polarization after brain trauma. *Neurotherapeutics* 11 679–695. 10.1007/s13311-014-0277-y 24965140 PMC4121458

