# Supplementary material for: A novel organotypic cortical slice culture model for traumatic brain injury: molecular changes induced by injury and mesenchymal stromal cell secretome treatment
Source: Front Cell Neurosci. 2023 Jul 18;17:1217987. doi: 10.3389/fncel.2023.1217987 (PMC10390737; doi:10.3389/fncel.2023.1217987)
Supplement: Supplementary file 1 [file Image_1.pdf]

Figure S1: Schematic drawing of the process used to obtain cortical brain slices.

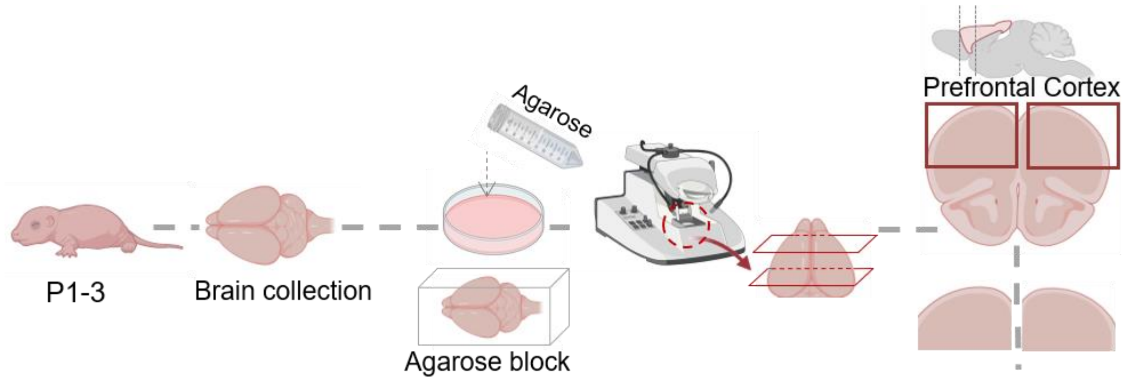

Image created with BioRender.com

Figure S2: Systems biology analysis of altered proteins which revealed several statistically significant molecular functions and biological process pathways

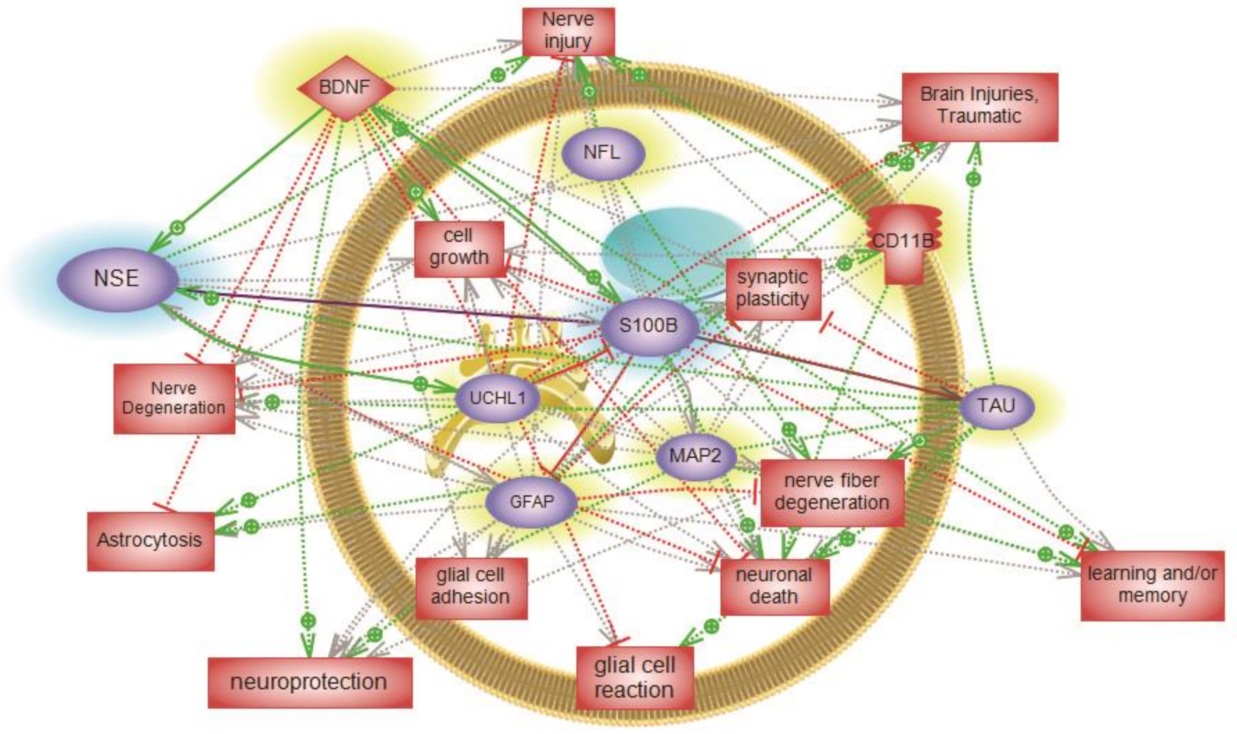

Pathway	p-value
Neurodegenerative Diseases	p<0.001
Nerve injury	p<0.001
Nerve fiber degeneration	p<0.001
Traumatic Injuries	p<0.001
Neuronal death	p<0.001
Learning and/or memory	p<0.001
Neuroprotection	p<0.001
Synaptic plasticity	p<0.001
Glial cell reaction	p<0.001
Astrocytosis	p<0.001
Cell growth	p<0.001
Glial cell adhesion	p<0.001

Picture shows the interaction map of altered protein and their corresponding relation to neural injury development; these statistically significant proteins include UCH-L1, GFAP, tau, Map2 (yellow hallow) as well as the two established TBI proteins S100B, and Neuron Specific Enolase (NSE) (Blue hallow).

Figure S3: Morphometric analysis of neuronal network after MSC-secretome treatment

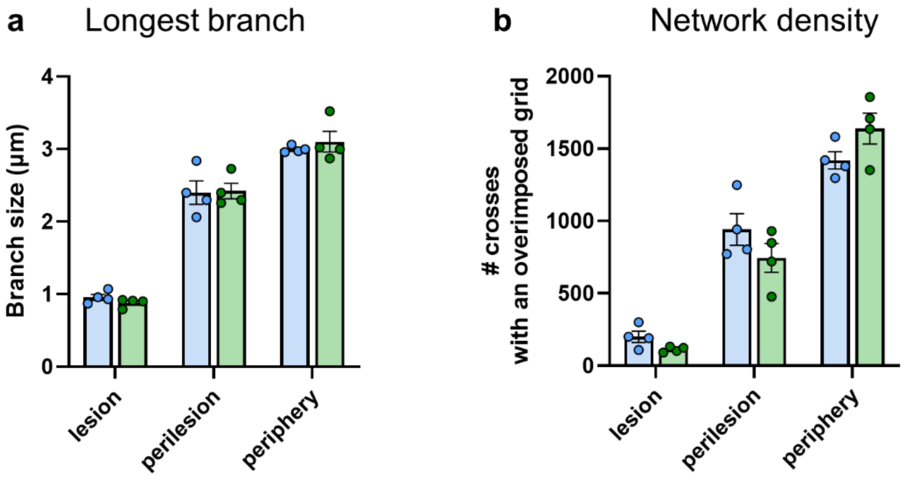

Quantification of the size of the longest branch (**a**) and the network density (**b**) of MAP-2 immunostained slices after MSC-secretome treatment. Data are mean ± SEM.

Figure S4: Microglial shape descriptors after MSC-secretome treatment.

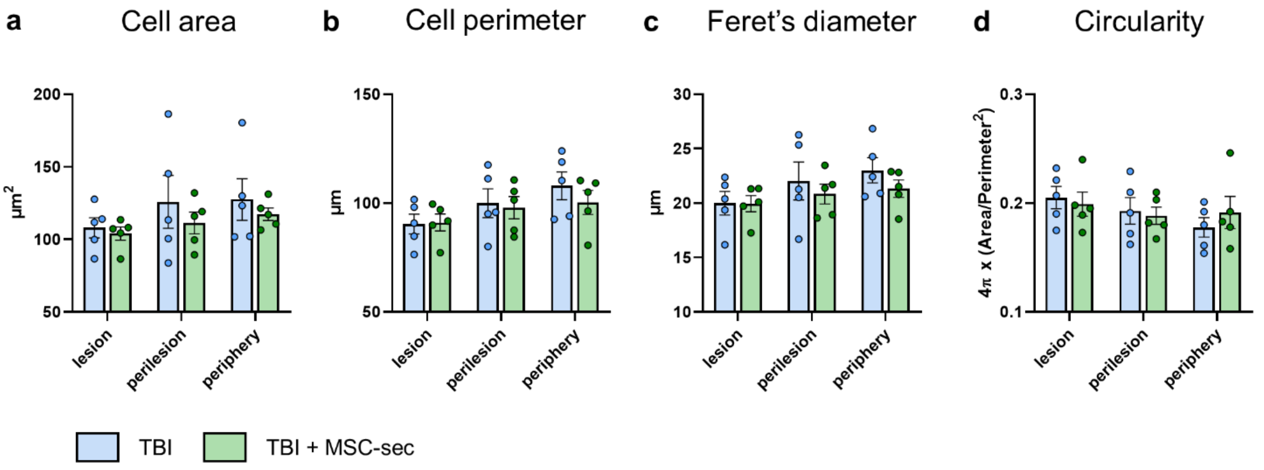

Quantification of morphometric microglia cell parameters after MSC-secretome treatment: mean cell area (**a**), perimeter (**b**), Feret's diameter (**c**) and circularity (**d**). Data are mean ± SEM.
